# HSV-2 Infection as a Potential Cofactor for HIV Disease Progression and Selection of Drug Resistance Mutations in Adults under WHO-Recommended First-Line Antiretroviral Therapy: A Multicentric, Cross-Sectional Study in Cameroon, Central African Republic, Chad, and Gabon

**DOI:** 10.3390/tropicalmed5030136

**Published:** 2020-08-24

**Authors:** Abdoulaye Mihimit, Chatté Adawaye, Hélène Péré, Cecilia Costiniuk, Donato Koyalta, François-Xavier Mbopi-Keou, Ralph-Sydney Mboumba Bouassa, Frédéric Talla, Sandrine Moussa, Jean De Dieu Longo, Bertin Hig-Zounet Tchombou, Gérard Grésenguet, Charlotte Charpentier, Laurent Bélec

**Affiliations:** 1 Ecole Doctorale d’Infectiologie Tropicale, BP: 876 Franceville, Gabon; mihimit2122@yahoo.fr (A.M.); ralphsydney87@yahoo.fr (R.-S.M.B.); 2 Centre Hospitalier Universitaire de la Mère et de l’Enfant, BP: 6667 N’Djamena, Chad; 3 Institut National Supérieur des Sciences et Techniques d’Abéché, BP: 130 Abéché, Chad; cadawaye@yahoo.fr; 4 Laboratoire de Microbiologie, Hôpital Européen Georges Pompidou, Assistance Publique-Hôpitaux de Paris, and Faculté de Médecine Paris Descartes, Université Paris Descartes, Sorbonne Paris Cité, 75015 Paris, France; helene.pere@aphp.fr; 5 Department of Medicine, Divisions of Infectious Diseases/Chronic Viral Illness Service, McGill University Health Centre, Montreal, QC H4A 3J1, Canada; cecilia.costiniuk@mcgill.ca; 6 Faculté des Sciences de la Santé Humaine, département de Médecine, Université de N’Djaména, BP: 1117 N’Djamena, Chad; koyaltad@yahoo.fr (D.K.); tchombouhzb@yahoo.fr (B.H.-Z.T.); 7 United Nations Programme on HIV and AIDS UNAIDS, BP: 1146 N’Djamena, Chad; 8 National Public Health Laboratory, Ministry of Public Health & University of Yaounde I, P.O. Box: 3601 Yaounde, Cameroon; fxmkeou@hotmail.com; 9 Laboratoire d’analyse médicales Litto-Labo, BP: 13038 Douala, Cameroon; littolabo@yahoo.fr; 10 Institut Pasteur de Bangui, BP:923 Bangui, Central African Republic; sandmoussa@hotmail.com; 11 Centre National de Référence des Maladies Sexuellement Transmissibles et de la Thérapie Antirétrovirale, BP:236 Bangui, Central African Republic; jddlongo@yahoo.fr; 12 Département de Santé Publique, Faculté des Sciences de la Santé de Bangui, Unité de Recherches et d’Intervention sur les Maladies Sexuellement Transmissibles et le SIDA, BP:923 Bangui, Central African Republic; gerardgres@yahoo.fr; 13 Laboratoire de Virologie, Hôpital Bichat-Claude Bernard, Assistance Publique-Hôpitaux de Paris, 75018 Paris, France; charlotte.charpentier@bch.aphp.fr

**Keywords:** HIV-1, HSV-2, surrogate markers, HIV-1 RNA load, drug resistance mutations, WHO recommendations, Central Africa

## Abstract

Although herpes simplex virus-2 (HSV-2) infection is a known cofactor for HIV transmission in Central Africa, its role in HIV disease progression is unclear. The aim of this study was to examine the potential link between HSV-2 infection and HIV disease progression, in addition to identifying the presence of genes conferring HIV antiretroviral resistance mutations. This was a cross-sectional study involving 302 HIV-infected adults in Central Africa with virological failure (viral load >1000 copies/mL) on first-line antiretroviral therapy from four different countries. The seroprevalence of HSV-2 was 32% (96/302). Amongst the HIV-infected individuals who were HSV-2 seropositive, the mean HIV viral load and CD4 count were 4.82 ± 0.83 log copies/mL and 243 ± 144 cells/microliter, respectively. Among the HIV-infected individuals who were HSV-2-seronegative, the mean HIV viral load and CD4 count were 3.48 ± 0.44 log copies/mL and 646 ± 212 cells/microliter, respectively (*p* < 0.001). There was a statistically significant relationship (*p* < 0.001) between HSV-2 seropositivity and the presence of resistance mutations to antiretrovirals (ARV), non-nucleoside reverse transcriptase inhibitors (NNRTI), and nucleoside reverse transcriptase inhibitors (NRTI) with odds ratios of 9.7, 10, and 11.9, respectively. There was no link between HSV-2 serostatus and protease inhibitor (PI) resistance mutations. There was a substantial accumulation of resistance mutations in HSV-2-seropositive compared to -seronegative patients. These findings support the link between HIV disease progression and HSV-2 infection. An association was observed between the presence of NNRTI and NRTI resistance mutations and HSV-2 seropositivity.

## 1. Introduction

Sub-Saharan Africa is the region of the world most profoundly affected by the HIV epidemic. Nearly 70% of all individuals infected with HIV live in Sub-Saharan Africa, although this region contains less than 12% of the world’s population. A major turning point in the HIV epidemic was the expansion of access to antiretroviral therapy (ART), with a concomitant reduction in the number of HIV-attributable deaths [1,2,3]. However, this new hope seems compromised by the appearance of HIV strains carrying antiretroviral resistance mutations [4].

Many factors may contribute to the emergence of antiretroviral-resistant HIV strains in Africa. These factors include insufficient patient follow-up and adherence monitoring [5,6], a shortage of medication available at pharmacies, resulting in treatment interruptions [7,8], and the existence of pre-existing antiretroviral resistance mutations [9,10]. Together, herpes simplex virus-2 (HSV-2) and HIV create a vicious circle, reinforcing the bidirectional interactions between each other [11,12]. HIV disease progression can be evaluated clinically or biologically with the aid of surrogate markers such as HIV viral load (a marker of rate of HIV disease progression) and CD4 T-cell count (a marker of HIV infection stage) [13]. Clinical progression is characterized by the appearance of opportunistic infection. Biological progression is characterized by a reduction in the CD4 T-cell count and a rise in the HIV plasma viral load [14]. Biological progress precedes clinical progression [15]. In Central Africa, the development of virological resistance was observed earlier than in other regions of the word [16]. In N’Djamena, an elevated prevalence (64%) of antiviral resistant strains was found in adults with HIV under first-line ART [17].

HSV-2 is a powerful co-factor facilitating heterosexual HIV-1 transmission in Central Africa [18,19,20,21]. Indeed, HIV-1 and HSV-2 entertain a bidirectional and complex synergistic relationship [22,23]. There are numerous molecular mechanisms by which HSV-2 can increase HIV-1 replication [22,24]. HIV-1 and HSV-2 are endemic to Central Africa. Interventional studies using acyclovir to treat HSV-2 infection demonstrated a clinical improvement in HIV and the slowing of HIV evolution [25,26,27]. Although somewhat controversial, some studies have demonstrated that individuals co-infected with HSV-2 and HIV could progress more rapidly in their illness [20,28]. Indeed, virological failure during the course of antiretroviral therapy affects nearly 35% of adults and 50% of children [17].

Data on the interaction between HSV-2 and HIV are lacking for Central Africa. Herein, we evaluate the interaction between HSV-2 and HIV by examining the potential association between HIV virological failure and HSV-2 co-infection in individuals under antiretroviral therapy according to the OMS recommendations. Specifically, we aimed to determine the presence of various resistance mutation genes for protease and reverse transcriptase in HIV-infected individuals under first-line ART and to describe the relationship between HSV-2 and the selection of antiretroviral resistance mutations. These findings could enable public health policies in Central African countries to systematically integrate the diagnosis of HSV-2 infection into strategies for managing people living with HIV, as a risk factor for therapeutic failure, rather than a simple opportunistic infection.

## 2. Material and Methods

### 2.1. Inclusion Sites and Study Population

**Figure 1:**
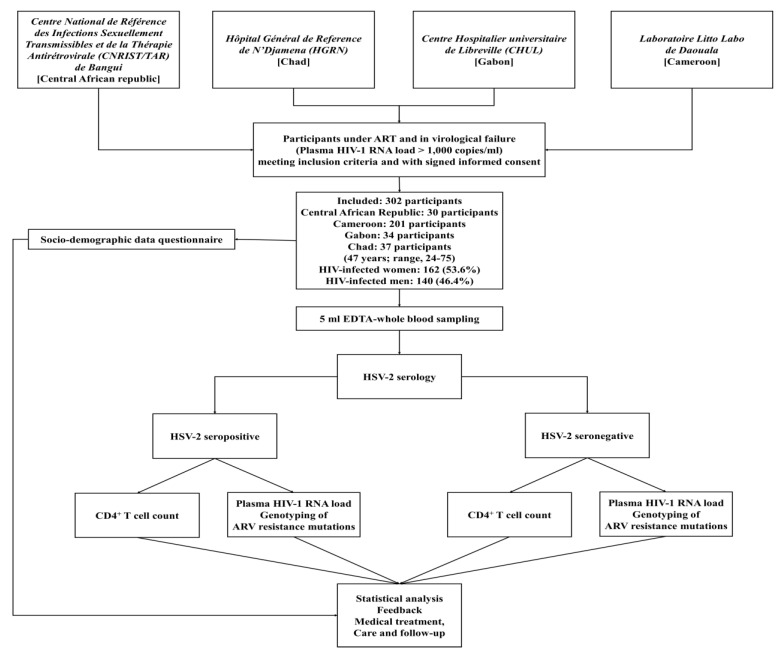
Flow-chart of the selection procedure of the study participants and inclusion sites.

The flow-chart of the Figure 1 summarizes the selection and inclusion process for study participants. In Central Africa, people living with HIV receive HIV and STI prevention and care at the “Centre National de Référence des Infections Sexuellement Transmissibles et de la Thérapie Antirétrovirale (CNRIST/TAR) de Bangui”, in Central African Republic, at the “Hôpital Général de Reference de N’Djamena (HGRN)”, Chad and at the “Centre Hospitalier Universitaire de Libreville (CHUL)” in Gabon and at the “Laboratoire Litto Labo de Daouala” in Cameroon. The main inclusion criteria for this study was the virological response of HIV-infected patients to ARV treatment. Thus, only patients with virological failure (HIV-1 RNA load > 1000 copies/mL) were included. These participants were then classified into two sub-groups based on HSV-2 seropositivity. One group was constituted with patients in virological failure to ARV and seropositive for HSV-2 and a second group was constituted with patients in virological failure to ARV, but seronegative to HSV-2 (Figure 1).

### 2.2. Collection of Biological Specimens

Specimens included plasma from 302 HIV-infected individuals with virological failure on first-line (OMS) antiretroviral therapy in four sites in Central Africa, Cameroon, Gabon, Central African Republic, and Chad, with 201, 34, 30, and 37 patients from each site, respectively. For all sites, inclusion criteria included: (1) being on first-line ART, as per OMS recommendations, for ≥6 months; (2) having virological failure, as determined by HIV viral load > 1000 copies/mL, as indicated by the revised OMS guidelines from July 2013, (3) providing informed consent to undergo routine and supplemental tests for monitoring. Exclusion criteria included (1) absence of accurate demographic and/or ART data; (2) age < 18 years. This study received ethical approval from the Minister of Public Health in Cameroon and Chad. Ethical approval was also received from the ethics committee of Gabon in addition to the scientific review committee of the Faculty of Health Sciences (Comité Scientifique Chargé de la Validation des Protocoles d’Etudes et des Résultats (CSCVPER)). Informed consent was obtained from all participants.

### 2.3. Monitoring Tests

For each participant, a CD4 T-cell count was performed, in addition to a plasma HIV viral load. Plasma viral load was measured using the NucliSENSEasyQ^®^ HIV-1 v2.0 (bioMérieux SA, Marcy L’Etoile, France) in Cameroon, branched DNA VERSANT^®^ HIV-1 RNA 1.0 kPCR (Siemens Healthcare Diagnostics, Malburg, Germany) in Gabon, Generic HIV Charge Virale (Biocentric, Bandol, France) in Central Africa, and the Abbott m2000rt Real Time™ HIV-1 assay (Abbott Laboratories, Chicago, IL, USA) in Chad. HSV-2 serology was performed using the HerpeSelect^®^ 2 ELISA IgG Herpes Simplex Virus-2 (HSV-2) ELISA IgG, Focus Technologies, Inc., Cypress, CA, USA [formerly MRL Diagnostics]).

### 2.4. Viral Subtype Determination

The viral subtype was derived from the FASTA format pol sequence using the Genotyping open-access software provided by the National Center for Biotechnology Information (National Center for Biotechnology Information, U.S. National Library of Medicine, 8600 Rockville Pike, Bethesda MD 20894 USA).

### 2.5. Genotyping for Antiretroviral Resistance Mutations

Genotyping for antiretroviral resistance mutations was performed in the virology laboratory at the George Pompidou European Hospital in Paris, using plasma aliquots conserved at −80 °C. Genotyping was performed using the technique from the resistance group of the French National Agency for AIDS Research [www.hivfrenchresistance.org]. Each sequence of interest was registered in GenBank. The viral subtype was deduced from sequences within the *pol* gene in FASTA format using the freely accessible genotyping software developed by the National Center for Biotechnology Information. Following gene sequencing of protease and reverse transcriptase, comparisons between amino acids in the sequences generated from patients and the reference sequences were made using the interpretation algorithms of resistance of the National Agency of AIDS Research and viral hepatitis of the ANRS 2013 (Agence Nationale de Recherche sur le Sida; 75,013, Paris, France) [http://www.hivfrenchresistance.org]. The list of mutations and their positions is updated regularly.

### 2.6. Statistical Analyses

Data were entered into an Excel table and analyzed using SPSS.18 and Epi Info.06.fr (ENSP-Epiconcept-InUS, 2001). A Chi-squared test was used to compare frequencies and the Fisher *t*-test was used to compare means with a 5% standard deviation. A measure of the association between variables was examined using odds ratios with 95% confidence intervals. To verify whether HSV-2 infection could constitute a risk factor associated with virological failure in patients infected with HIV in Central Africa, we made the following null hypothesis: there is no significant (*p*-value > 0.05) difference in virological (plasma HIV-RNA load, ARV resistance mutations and level of circulating genetic diversity) and immunological (CD4+ T cell count) parameters between HSV-2 seropositive and HSV-2 seronegative individuals.

## 3. Results

### 3.1. Study Population

A total of 302 HIV-infected individuals with virological failure were included in this study, including 201 from Cameroon, 34 from Central African Republic, 30 from Gabon, and 37 from Chad (Table 1). The mean age was 47 ± 10 (24–75) years for all individuals. The mean age was slightly higher for individuals from Cameroon at 48 ± 9 years followed by Central Africa at 46 ± 8 years, Gabon at 45 ± 10 years, and Chad at 41 ± 10 years. There were no significant differences between mean participant age across the various countries. Women comprised 162 out of the 302 participants. There was no significant difference in the proportions of women across the groups. The mean duration of treatment was 41 ± 33 months with a range of 6‒141 months. A statistically significant difference in the mean duration was noted between the countries. The mean viral load was 3.91 ± 0.86 Log copies/mL (range 3.00 to 6.24 Log copies/mL). The mean CD4 T-cell count was 518 ± 269 cells/µL (range 12 to 1112 cells/µL). A statistically significant difference was noted between countries, with lower mean CD4 T-cell counts in participants in Central Africa and Chad. HSV-2 serology was positive in 96 out of 302 participants (32%). There was no statistically significant difference between the proportions of participants who were HSV-2 co-infected between the countries.

### 3.2. Genetic Diversity of HIV-1 Variants in Central Africa

**Figure 2:**
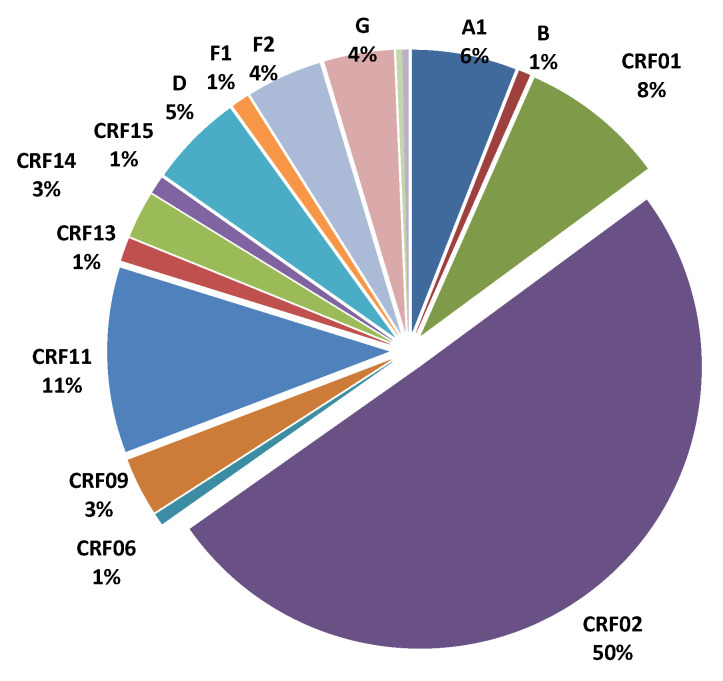
Distribution of virus subtypes in 302 patients with virological failure in Central Africa.

The genetic diversity of the HIV-1 variants of the selected population is shown in Figure 2. There was a slight predominance of CRF02, accounting for 50% of viral strains.

**Figure 3:**
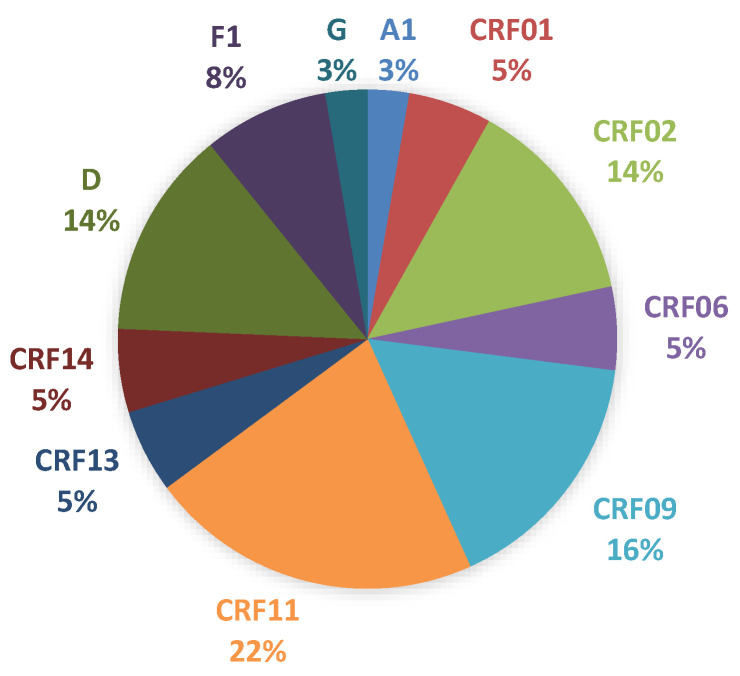
Distribution of virus subtypes in 37 patients with virological failure in Chad.

We will focus on the genetic diversity found in Chad. Chad is home to remarkable genetic diversity (Figure 3).

### 3.3. HSV-2 Serology and HIV Disease Progression

The mean viral load of HIV-1 was higher and mean CD4 T lymphocytopenia was lower in seropositive patients for HSV-2 compared to those seronegative for HSV-2 with a statistically significant difference (Table 2). A difference was observed in samples from all Central African countries of the study. The increase in viral load and the decrease in TCD4 lymphocyte count as markers of HIV.

**Figure 4:**
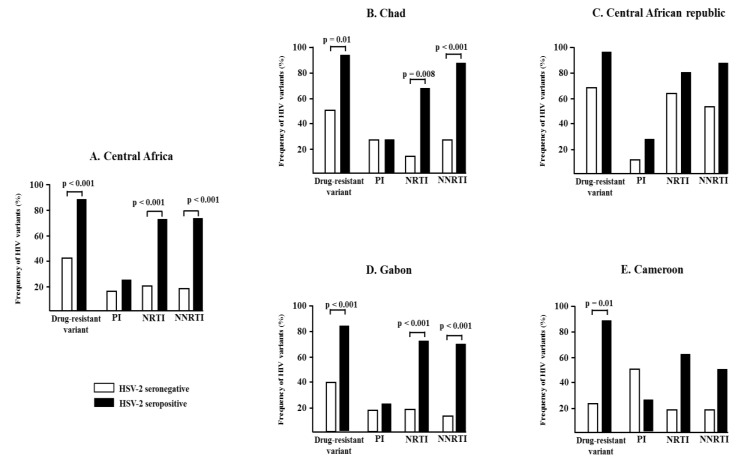
Frequency of ARV resistance mutations according to HSV-2 seropositivity.

The Figure 4 depicts the proportions of antiretroviral-resistant variants among HIV-1 infected individuals who are HSV-2-seropositive versus those who are HSV-2-seronegative. The prevalence of antiretroviral-resistant variants (NNRTI: non-nucleoside reverse transcriptase inhibitors; NRTI: nucleoside reverse transcriptase inhibitors) was higher amongst HSV-2 seropositive individuals versus those who were HSV-2-seronegative, except in Gabon and the Central African Republic. The prevalence of antiretroviral resistance mutations to protease inhibitors was similar among HSV-2-seropositive versus HIV-2-seronegative individuals. Globally, the odds ratio for finding antiretroviral resistance variants among individuals who were HSV-2-seropositive was 9.7. The odds ratio of having NNRTI-resistant variants was 10.2 in the case of HSV-2 seropositivity and 11.9 for NRTI-resistant variants in the case of HSV-2 seropositivity.

### 3.4. HSV-2 Serology and Selection of Resistance Mutations in HIV-1 Protease and Reverse Transcriptase Genes

The proportions of mutations observed in the reverse transcriptase gene between HIV-infected individuals who were seropositive for HSV-2 vs. seronegative for HSV-2 were most often significant, except for positions V90I K103H/N/S/T, K103R, V106A/I/M, E138A/G/Q/R/, V179L, V179D/F/I/M, and Y181C/I, where no significant difference was observed (Table 3). 

The proportion of mutations observed in the protease gene in HSV-2-seropositive individuals vs. -seronegative ones was often not significantly different, except for positions I15A/L/V, E34A/G/K, M46I/L/V, and V82A/F/M/S/T, where the differences were significant (Table 4): HSV-2 seropositivity and the selection of antiretroviral resistance mutations in the protease gene in 302 HIV-infected individuals on first-line treatment with virological failure in Central Africa are shown in Table 4.

## 4. Discussion

### 4.1. Concerning the Population Studied

The average age was 47 years old for all countries. There was no statistically significant difference in age averages between the study countries, ensuring the possible comparability of the cohorts included in each country. There is a predominance of women in the cohorts studied, with no differences between the four countries of study. The predominance of women in therapeutic cohorts is classic in Africa south of the Sahara and indirectly reflects the vulnerability of women to HIV transmission. 

The seroprevalence for anti-HSV-2 antibodies in our study was 32%. This prevalence approaches that observed by other authors in the subregion. Furthermore, an investigation of the seroprevalence of HSV in people living with HIV in Cameroon demonstrated a rate of 22% HSV-2 and 34% HSV-2/HSV-1 in a sample consisting of 100 adults [29]. In contrast, the seroprevalence of HSV-2 in our series was less than that reported in a study conducted in Gabon, where a seroprevalence of 66% was documented among 355 pregnant women receiving maternity care [30]. Another study in South Africa similarly showed a prevalence of 66% [31]. This difference could be attributed to the difference in sex ratio of the participants included in the series. Indeed, herpes is more easily transmitted to women than to men. The cohorts included in the study performed in Gabon [30] and in South Africa [31] were comprised uniquely of women, whereas our study cohort consisted of both men and women.

Nevertheless, these seroprevalences confirm the high prevalence of herpes in Central Africa, and the high prevalence of persons co-infected with HIV-1 and herpes, two chronic viral illnesses.

### 4.2. Genetic Diversity of HIV-1 Strains

In our series, there is a great genetic diversity of HIV-1 subtypes. We focus particularly more on the genetic diversity found in Chad. The distribution of HIV-1 subtypes in Chad is unique in the region for several reasons: a high prevalence of CRF1l_cpx (probably nearly a quarter of infections), a high prevalence of subtype D, and low proportions of CRF02_AG and CRF06_cpx. The work of Vidal [32] and Koyalta [17] showed the circulation of three major subtypes (in order of frequency: A, D, and G) and four CRPs with a predominance of CRF1l_cpx, then CRF02_AG, CRF01_AE, and CRF06_cpx. Subtype A is extensively involved in various recombinations with other variants, often CRFs, causing complex recombinant forms, and Vidal identified 12 different profiles (Al/CRFll_cpx, A1/CRF02_AG, Al/D, and Al/CRF01_AE, for example) [32]. Second-generation secondary recombinants derived from the recombination of two CRFs have also been characterized (CRF02_AG/CRF11_cpx, CRF01_AE/CRFll_cpx). A small proportion of isolates (approximately 3%) remain unclassifiable after phylogenetic analysis of the envV3-V5 and gagp24 fragments [32]. Interestingly, the subtype D strains originating from Chad form a phylogenetic line that is individualized within the other D variants characterized in Africa. East Africa or elsewhere in Central Africa, however, after complete sequencing of the genome, strains of this phylogenetic subgroup do not meet the criteria for defining a sub-subtype within subtype D [32]. It is, therefore, a geographical variant that reflects a country-specific founding effect. Finally, the Kayolta study [17] leads us to add a remark. In this prospective study evaluating the efficacy of antiretroviral therapy at six months in 88 first-line patients, only 47% achieved an undetectable viral load at six months. The persistence of viral replication was associated with the presence of at least one resistance mutation in two-thirds of patients, which highlights the operational difficulties involved in dispensing antiretrovirals in a country where the inadequacy of the health system is a major issue [17].

### 4.3. HSV-2 Serology and HIV Disease Progression

Our study enabled us to demonstrate an association between HSV-2 seropositivity and biological markers of HIV disease progression (*p* < 0.001). Furthermore, persons who were seropositive for HSV-2 had a mean CD4 T-cell count that was lower (243 ± 144 par per mm^3^) than that observed in those who were HSV-2-seronegative (646 ± 212 par per mm^3^) and a higher mean HIV viral load (4.82 ± 0.83 log copies/mm^3^) than in those who were HSV-2 seronegative (3.48 ± 0.44 log copies/mm^3^).

These observations were in contrast with those from the study by Roxby et al. [33] performed in Kenya, involving 296 postpartum HIV-1-infected women, wherein no statistically significant difference was found between the rate of CD4 T-cell lymphopenia <200 par per mm^3^ and HSV-2 seropositivity. However, the bias of this former study was probably the utilization of acyclovir in patients who may have been infected by herpes. Furthermore, the work by Mohraz et al. 2018 [34] in Iran in antiretroviral-naive patients followed for a year did not document a statistically significant difference in the CD4 T-cell counts or HIV viral loads in HSV-2-seropositive versus HSV-2-seronegative individuals. Different findings between the studies could be explained by the size of the specimen and especially, the number of HSV-2-seropositive persons in the cohorts. In contrast, our results are consistent with those of other studies conducted in Sub-Saharan African. Furthermore, Anoma et al. [35] carried out a study in the Ivory Coast involving 132 prostitutes with HIV infection and demonstrated that the average HIV viral load was higher in women who had genital ulcers due to either herpes or *Haemophilus ducreyi* compared to women whose genital ulcers were due to an unclear etiology (HIV viral load 3.2 log copies/mL for women with HSV-2 infection; 3.3 log copies/mL for women with *Haemophilus ducreyi*; 4.6 log copies/mL for women co-infected with HSV-2 and *Haemophilus ducreyi*, vs. 2.5 log copies/mL pour women whose ulcers were of indeterminate etiology; *p* = 0.04). In two studies in Uganda [36,37] consisting of 339 and 256 individuals, respectively, persons co-infected with HSV-2 and HIV had a higher mean HIV viral load than those who were HIV-1 mono-infected, a difference that was statistically significant (*p* = 0.014 and *p* < 0.001, respectively).

Similarly, Looker et al. [38] found a high viral load of HIV attributable to HSV-2 infection according to a meta-analysis relating to six studies worldwide.

The higher plasma HIV viral loads in persons with HIV and HSV-2 co-infection may be the consequence of bidirectional interactions and synergies which exist between HIV and HSV. Indeed, it has been shown that numerous proteins expressed by HSV, products of early gene expression, are capable of transactivating HIV replication in vitro by interacting with the regulating HIV *Long Terminal Repeat* region [39].

Resistance to antiretroviral drugs is a consequence of the accumulation of resistance mutations that are found on the regions of the genes on which antiretroviral drugs exert their action. Our study enabled us to study the association between the presence of resistance mutations on the protease and the reverse transcriptase genes based on the HSV-2 serological status. Furthermore, 88% of people seropositive for HSV-2 had an HIV resistance mutation to at least one antiretroviral vs. 42% of HSV-2-seronegative people, with a difference that statistically significant (*p* < 0.001) and an odds ratio of 9.7 [5; 20]. The association between HSV-2 seropositivity and the selection of resistance mutations was more important for NNRTI and NRTI with odds ratios of 10.2 [5.6; 18.2] and 11.9 [6; 4; 2; 0], respectively. In contrast, there was no significant difference with protease inhibitors (PI). It must be underscored that all persons included in this analysis were experiencing virological failure while on first-line therapy suggested by the OMS, for which the regimen includes two NRTI and one NNRTI. The PI are reserved for second-line treatment in Central Africa [40,41].

The selection of the most important antiretroviral-resistant variants to first-line therapy could be the consequence of HIV and HSV-2 co-infection, which appears to boost HIV-1 replication in the presence of antiretroviral-induced selection pressure.

The gene regions conferring antiretroviral resistance mutations among persons with HIV-HSV-2 co-infection with a statistically significant difference versus those who were HSV-2-seronegative were as follows. For NNRTI, the A98G/S mutation confers resistance to nevirapine; the K103H/N/S/T mutation confers to resistance nevirapine, efavirenz, and/or rilpinavir; and the Y181V mutation confers major resistance to rilpinavir and etravirine. This last mutation would be due to a prior polymorphism as rilpinavir and etravirine are new molecules that are not used in current practice in first-line treatment in Central Africa; finally, the mutations M230I/L/V and Y188C/H/L confer major resistance to nevirapine and efavirenz, the principal antiretrovirals used as first-line therapy. K103N and Y181C confer irreversible resistance to efavirenz and nevirapine. For NRTI, M41L, D67N, T69D/N/S, and K219Q mutations, which are minor mutations, and QI51/M and M184V, which are major mutations, confer cross-resistance to two NRTIs. The M184V mutation is the mutation most often observed in the thymidine analogue mutations (TAMs), found in 68% of persons seropositive for HSV-2. This mutation is the mutation most rapidly selected out. For the PI, the majority of mutations observed did not have a statistically significant difference, which may be explained by the fact that the PI are not generally used in HIV-infected persons in first-line therapy in Central Africa. The only mutations for which there was a statistically significant difference between persons seropositive for HSV-2 vs. seronegative for HSV-2 (I15A/L/V, E34A/G/K, M36I/L/V, and V82A/F/M/S/T) constituted mutations linked to polymorphism.

Although we provide here relevant data demonstrating the association between HSV-2 seropositivity and the severity of HIV-infection across Central Africa, our study had some limitations. Indeed, as we were focused particularly on virologic (HIV-1 viral load and accumulation of DRMs) and immunologic (significant reduction of CD4+ T cells counts) markers to assess the disease progression, we did not address whether HIV disease progression observed would not also be associated with other risk factors such as co-infection with opportunistic pathogens which could over stimulate the immune system leading to a generalized inflammation therefore worsening the HIV-infection. 

## 5. Conclusions

Our study, conducted with 302 HIV-infected individuals with virological failure under first-line therapy in four countries in Central Africa, enabled us to demonstrate an association between HSV-2 seropositivity and HIV disease progression, as measured by increased HIV plasma viral load and reduction in CD4 T-cell count compared to those who were HSV-2-seronegative. Furthermore, our study demonstrated an association between the selection of HIV variant antiretroviral resistance mutations and HSV-2 infection. The odds ratio was most pronounced for NNRTIs and NRTI, but there was no significant difference for PIs. Our study also enabled us to demonstrate the association between HSV-2 and the type of antiretroviral resistance mutations selected out on the reverse transcriptase gene. This study enabled us to demonstrate that individuals who were HSV-2-seronegative had a lower mutation and therefore a lower risk of selecting out HIV with antiretroviral resistance, with a statistically significant difference; hence, the management of herpes could be part of the strategy of preventing HIV resistance to antiretroviral drugs.

## Figures and Tables

**Table 1:** Characteristics of patients under first-line antiretroviral treatment experiencing virological failure in Central Africa.

Characteristics of Patients	Total	Cameroon	Central African Republic	Gabon	Chad	*p*
Number	302	201	34	30	37	
Age (years, mean ± σ)	47 ± 10	48 ± 9	46 ± 8	45 ± 10	41 ± 10	*NS*
Sex (*n*, (%))						
✓ Men	116 (38)	73 (36)	12 (35)	11 (37)	20 (54)	NS
✓ Women	186 (62)	128 (63)	22 (65)	19 (63)	17 (46)	NS
Duration de antiretroviral treatment	-	-	-	-	-	-
(months, mean ± σ)	41 ± 33	50 ± 35	14 ± 13	23 ± 15	28 ± 25	<0.001
HIV viral load (log copies/mL, mean ± σ)	3.91 ± 0.86	3.89 ± 0.89	4.12 ± 0.62	3.71 ± 0.84	4.00 ± 0.85	0.028
CD4 T-cells (/µL, mean ± σ)	518 ± 269	537 ± 275	420 ± 235	578 ± 259	455 ± 259	<0.04
HSV-2 seropositivity (*n*, (%))	96 (32)	58 (29)	15 (44)	8 (17)	15 (40)	NS
Antiretroviral resistance variants (*n*, (%))	170 (56)	104 (52)	28 (82)	13 (43)	25 (68)	0.002
Antiretroviral resistance variants to PI (*n*, (%))	56 (19)	37 (18)	6 (18)	3 (10)	10 (27)	NS
Antiretroviral resistance variants to NNRTI (*n*, (%))	110 (26)	67 (33)	24 (70)	25 (17)	19 (51)	<0.001
Antiretroviral resistance variants to NRTI (*n*, (%))	109 (25)	58 (29)	23 (67)	8 (29)	14 (38)	<0.001

σ: standard deviation; PI: protease inhibitors; NNRTI: non-nucleoside reverse transcriptase inhibitors; NRTI: nucleoside reverse transcriptase inhibitors; NS: nonsignificant.

**Table 2:** HSV-2 seropositivity, HIV-1 viral load, and CD4 T-cell count in 302 individuals under first-line therapy with virological failure in Central Africa (Cameroon, Central African Republic, Chad, and Gabon).

	*n*	HSV-2 Seronegative (*n*, (%)]	HSV-2 Seropositive (*n*, (%)]	*p*
**Cameroon**	201	143 (71)	58 (29)	
HIV viral load (mean ± σ) *		3.47 ± 0.45	4.90 ± 0.91	<0.001
CD4 T-cell count (mean ± σ) **		656 ± 217	243 ± 161	<0.001
**Gabon**	30	22 (73)	8 (27)	
HIV viral load (mean ± σ) *		3.33 ± 0.24	4.75 ± 1.02	<0.001
CD4 T-cell count (mean ± σ) **		702 ± 161	236 ± 135	<0.001
**Central African Republic**	34	19 (56)	15 (44)	
HIV viral load (mean ± σ) *		3.72 ± 0.44	4.63 ± 0.41	<0.001
CD4 T-cell count (mean ± σ) **		551 ± 219	255 ± 128	<0.001
**Chad**	37	22 (59)	15 (41)	
HIV viral load (mean ± σ) *		3.48 ± 0.45	4.75 ± 0.74	<0.001
CD4 T-cell count (mean ± σ) **		605 ± 217	255 ± 128	<0.001
**TOTAL**	302	206 (68)	96 (32)	
HIV viral load (mean ± σ) *		3.48 ± 0.44	4.82 ± 0.83	<0.001
CD4 T-cell count (mean ± σ) **		646 ± 212	243 ± 144	<0.001

* HIV viral load is expressed Log copies/mL, mean ± σ; ** CD4 T-cell count is measured is expressed as cells/µL of blood, mean ± σ; σ: standard deviation.

**Table 3:** HSV-2 seropositivity and selection of antiretroviral resistance mutations in the reverse transcriptase gene.

	HSV-2 Seronegative *n* = 206 (*n* (%))	HSV-2 Seropositive *n* = 96 (*n* (%))	*p*	OR (IC_95%_) ^£^
**Mutations to NNRTI**				
V90I	9 (4)	5 (5)	NS	-
A98G/S	4 (2)	15 (16)	<0.001	9.5 (2.8; 34.4)
L100I	0 (0)	3 (3)	NS	-
K101H/I/R	4 (2)	19 (20)	<0.001	12.4 (3.8; 44.8)
K103H/N/S/T	20 (9)	37 (38)	<0.001	5.8 (3.0; 11.3)
K103R	1 (1)	2 (2)	NS	-
V106A/I/M	8 (4)	8 (8)	NS	-
E138A/G/Q/R/S	9 (4)	5 (5)	NS	-
V179L	16 (8)	13 (14)	NS	-
V179D/F/I/M/T	6 (3)	1 (1)	NS	-
Y181C/I	8 (4)	0 (0)	NS	-
Y181V	1 (1)	11 (12)	<0.001	26.5 (3.5; 55.8)
Y188C/H/L	1 (1)	4 (4)	NS	
G190A/S	5 (2)	15 (16)	<0001	7.4 (2.4; 24.3)
G190C/E/Q/T/VH221Y	0 (0)	0 (0)	-	
P225H	3 (1)	9 (9)	0.003	7.0 (1.7; 23.2)
M230I/L/V	3 (3)	0 (0)	NS	-
P236L	0 (0)	0 (0)	-	-
**Mutations to NRTI**				
M41L	3 (1)	14 (15)	<0.001	11.5 (3.0. 52.0)
E44D	0 (0)	7 (7)	<0.001	-
K65R	1 (1)	2 (2)	NS	-
D67N	6 (3)	19 (20)	<0.001	16.7 (4.5; 73.1)
T69D/N/S	5 (2)	11 (12)	<0.001	5.2 (1.6; 17.7)
Insertion 69	0 (0)	0 (0)	-	
K70E/R	8 (4)	20 (21)	<0.001	6.5 (2.4; 11.7)
L74V/I	0 (0)	0 (0)	-	-
V75A/M/S/T	4 (2)	10 (10)	0.003	5.8 (1.6; 22.9)
F77L	1 (1)	3 (3)	NS	-
Y115F	1 (1)	1 (1)	NS	-
Q151M	1 (1)	6 (6)	0.02	13.6 (1.6; 305)
M184V	31 (15)	64 (67)	<0.001	11.3 (6.1; 20.8)
L210W	3 (2)	6 (6)	0.055 NS	
T215Y/F	11 (4)	28 (29)	<0.001	7.3 (3.2; 7.5)
T215A/C/D/E/G/H/I/L/S/	2 (1)	5 (5)	NS	
K219Q/E	5 (2)	13 (14)	<0.001	6.3 (2,0;15,2)

NNRTI: non-nucleoside reverse transcriptase inhibitors; NRTI: nucleoside reverse transcriptase inhibitors; NS: nonsignificant; ^£^ OR (CI_95%_): odds ratio (95% confidence interval).

**Table 4:** HSV-2 seropositivity and selection of antiretroviral resistance mutations in the protease gene.

	HSV-2 Seronegative *n* = 206 (*n*, (%)]	HSV-2 Seropositive *n* = 96 (*n*, (%))	*p*	OR (IC_95%_) ^£^
Protease Inhibitor Resistance Mutations				
L10F/I/L/M/R/V	72 (35)	39 (40)	NS	-
V11I	8 (4)	3 (3)	NS	-
I15A/L/V	40 (19)	33 (34)	0.004	2.2 (1.2; 3.9)
G16A/E	57 (28)	29 (30)	NS	-
K20I/M/R/T	150 (73)	69 (72)	NS	-
L24I	0 (0)	1 (1)	NS	-
L33F/I/V	3 (1)	2 (2)	NS	-
E34A/G/K	11 (5)	0(0)	0.048	0.0 (0.0; 0/9)
M36I/L/V	192 (93)	87 (91)	NS	
M46I/L/V	9 (4)	11 (11)	(0.02)	2.8 (1.1; 7.7)
I47V/A	0 (0)	0 (0)	-	-
G48V	0 (0)	0 (0)	-	-
I50L/V	0 (0)	0 (0)	-	-
F53L/W/Y	2 (1)	0 (0)	NS	-
I54V/A/S/T/L/M	0(0)	5 (5)	0.05	-
Q58E	3 (1)	3 (3)	NS	-
D60E	32 (15)	17 (18)	NS	-
I62V	34 (16)	13 (13)	NS	-
L63P/V	111 (53)	48 (50)	NS	-
H69K/N/Q	189 (92)	84 (87)	NS	-
A71V/T/I/L	6 (2)	5 (5)	NS	-
G73S/T/C/A/V	1 (1)	0 (0)	NS	-
T74P	4(2)	5 (5)	NS	-
L76V	3 (2)	3 (3)	NS	-
V82A/F/M/S/T	16 (8)	15 (16)	0.036	2.2 (0.9; 4.9)
I84V	1 (1)	2 (2)	NS	-
I85V	3 (2)	2 (2)	NS	-
N88S/D	0 (0)	1 (1)	NS	-
L89I/M/R/T/V	184 (89)	81 (84)	NS	-
L90M	4 (2)	5 (5)	NS	-

PI: protease inhibitors; NS: nonsignificant; ^£^ OR [CI_95%_]: odds ratio [95% confidence interval].
